# Bone density optimized pedicle screw insertion

**DOI:** 10.3389/fbioe.2023.1270522

**Published:** 2023-10-26

**Authors:** Christos Tsagkaris, Anna-Katharina Calek, Marie-Rosa Fasser, José Miguel Spirig, Sebastiano Caprara, Mazda Farshad, Jonas Widmer

**Affiliations:** ^1^ Department of Orthopedics, Balgrist University Hospital, University of Zurich, Zurich, Switzerland; ^2^ Spine Biomechanics, Department of Orthopedic Surgery, Balgrist University Hospital, University of Zurich, Zurich, Switzerland; ^3^ Institute for Biomechanics, ETH Zurich, Zurich, Switzerland; ^4^ University Spine Center Zurich, Balgrist University Hospital, University of Zurich, Zurich, Switzerland

**Keywords:** pedicle screw, trajectory, spine, surgical planning, spine biomechanics

## Abstract

**Background:** Spinal fusion is the most common surgical treatment for the management of degenerative spinal disease. However, complications such as screw loosening lead to painful pseudoarthrosis, and are a common reason for revision. Optimization of screw trajectories to increase implant resistance to mechanical loading is essential. A recent optimization method has shown potential for determining optimal screw position and size based on areas of high bone elastic modulus (E-modulus).

**Aim:** The aim of this biomechanical study was to verify the optimization algorithm for pedicle screw placement in a cadaveric study and to quantify the effect of optimization. The pull-out strength of pedicle screws with an optimized trajectory was compared to that of a traditional trajectory.

**Methods:** Twenty-five lumbar vertebrae were instrumented with pedicle screws (on one side, the pedicle screws were inserted in the traditional way, on the other side, the screws were inserted using an optimized trajectory).

**Results:** An improvement in pull-out strength and pull-out strain energy of the optimized screw trajectory compared to the traditional screw trajectory was only observed for E-modulus values greater than 3500 MPa cm^3^. For values of 3500 MPa cm^3^ or less, optimization showed no clear benefit. The median screw length of the optimized pedicle screws was significantly smaller than the median screw length of the traditionally inserted pedicle screws, *p* < 0.001.

**Discussion:** Optimization of the pedicle screw trajectory is feasible, but seems to apply only to vertebrae with very high E-modulus values. This is likely because screw trajectory optimization resulted in a reduction in screw length and therefore a reduction in the implant-bone interface. Future efforts to predict the optimal pedicle screw trajectory should include screw length as a critical component of potential stability.

## 1 Introduction

Spinal fusion, which typically consists of a combination of posterolateral pedicle screw instrumentation and rod construct (Verlaan et al., 2004; Mirza and Deyo, 2007), is the most common surgical treatment modality for the management of degenerative spinal disease. A large number of studies have shown that spinal fusion procedures have a positive impact on patient outcomes (Reisener et al., 2020). However, short-to long-term complications such as screw loosening and implant failure lead to pseudoarthrosis, which can be a cause of pain, and is a common reason for revision surgery (Tokuhashi et al., 2008; Kim et al., 2020). Screw loosening rates of 10%–60% have been reported in the literature (Ohlin et al., 1994; Abul-Kasim and Ohlin, 2014; Galbusera et al., 2015; Bredow et al., 2016; Zou et al., 2019; Kim et al., 2020; Bokov et al., 2021), with higher rates in motion-preserving constructs and in patients with osteoporosis (Saman et al., 2013; Weiser et al., 2017). Implants’ material and their microscopic design have also been shown to be a risk factor for screw loosening in the absence of sufficient gripping and frictional resistance against the counter-movement of the instrumented vertebrae (Patel et al., 2015).

Computer-aided preoperative planning approaches have the potential to improve surgical outcomes by analyzing patient-specific three-dimensional (3D) key aspects. With rapid technological improvements in simulation models and intraoperative navigation in recent years, customized surgical plans can be created and implemented (Taylor and Prendergast, 2015; Vávra et al., 2017; Esfandiari et al., 2018; Farshad et al., 2022). A number of studies have already addressed screw positioning optimization methods that attempt to reduce screw loads and define optimized trajectories that improve screw retention using geometric futures of the vertebrae extracted from imaging obtained for the sake of the standard preoperative workup and planning (Solitro and Amirouche, 2016; Knez et al., 2019; Caprara et al., 2022). Recently, Caprara et al. (Caprara et al., 2022) developed an optimization method combining a genetic algorithm (GA) method and finite element (FE) analysis to provide an automated system for determining optimal screw position and size. The implementation used a combination of input parameters to maximize the mechanical properties of the vertebral bone within the simplified volume of the screw. GA performance was evaluated by comparing the screw positioning to the clinical standard. Overall, the optimization of screw trajectory and screw size, which was based on a computer simulation, resulted in a 26% increase in pull-out strength compared to conventional screw trajectory.

The aim of this biomechanical study was to verify the optimization algorithm for pedicle screw placement developed by Caprara et al. (Caprara et al., 2022) in a cadaveric study and to quantify the effect of optimization. For this purpose, the pull-out strength of pedicle screws with an optimized trajectory was compared to that of a traditional trajectory.

## 2 Materials and methods

The study was approved by the local ethics committee. Twenty-five lumbar vertebrae (five L1, five L2, five L3, five L4, five L5) obtained from five fresh-frozen cadavers (Science Care, Phoenix, AZ, United States) were tested in this study. The median age was 60 years (range 47–75 years, three males and two females).

### 2.1 Genetic algorithm optimization

The optimization algorithm used for screw positioning was described in detail in a previous study (Caprara et al., 2022). In brief, the vertebrae were segmented from the CT images of the five lumbar spines. Deformable 3D template models were used to determine the starting points for GA optimization. The template was non-rigidly registered to the segmented model in the Scalismo package (University of Basel, Switzerland) using an image registration point set (Clogenson et al., 2015). Using labeled regions on the template, it was possible to determine the entry point with respect to the segmented spine model after non-rigid registration. The pedicles could be automatically identified based on the vertebral endplates and correspondence properties of the template model after registration. For the GA procedure, the original CT image, the screw entry points, and a 3D grid of uniformly distributed points within the pedicles were used as the initial population. For each considered combination of entry point, pedicle points, screw length, and screw diameter, a cylinder of screw dimensions was placed in the vertebral body model. The fitness function used for performance assessment consisted of the sum of the elastic modulus (E-modulus) transformed CT voxel intensities within the cylinder (Keller, 1994; Rho et al., 1995). The latter consists of a measure of the resistance of the material, in particular vertebral bone tissue, to elastic deformation. The clinical feasibility of the pedicle screw position was ensured by setting two constraints. The first constraint guaranteed that the screw would be positioned completely within the vertebra and would not perforate the bone except at the screw head towards the insertion point. The second constraint forbade screw trajectories from crossing the sagittal midplane of the vertebral body (i.e., restricting each screw to either the left or the right half and therefore avoiding impracticable implant overlaps).

An experienced spine surgeon used the MySpine planning software (Medacta SA International, Switzerland) to define the standard screw trajectory for the pedicles considered. The default positions were then used to place simplified screws in the 3D vertebral body models and initialize the GA method for all segmented vertebrae. The labeled entry points, pedicle points, and available screw lengths and diameters were used as input for the optimization to create the initial population for the genetic algorithm. Screw lengths varied from 25 to 60 mm (5 mm increments) and screw diameters varied from 5 to 7 mm (1 mm increments). The dimensions were selected from the MUST (Medacta universal Screw Technology) pedicle screw system (Medacta International SA, Castel San Pietro, Switzerland).

### 2.2 Biomechanical experiments and testing protocol

**FIGURE 1:**
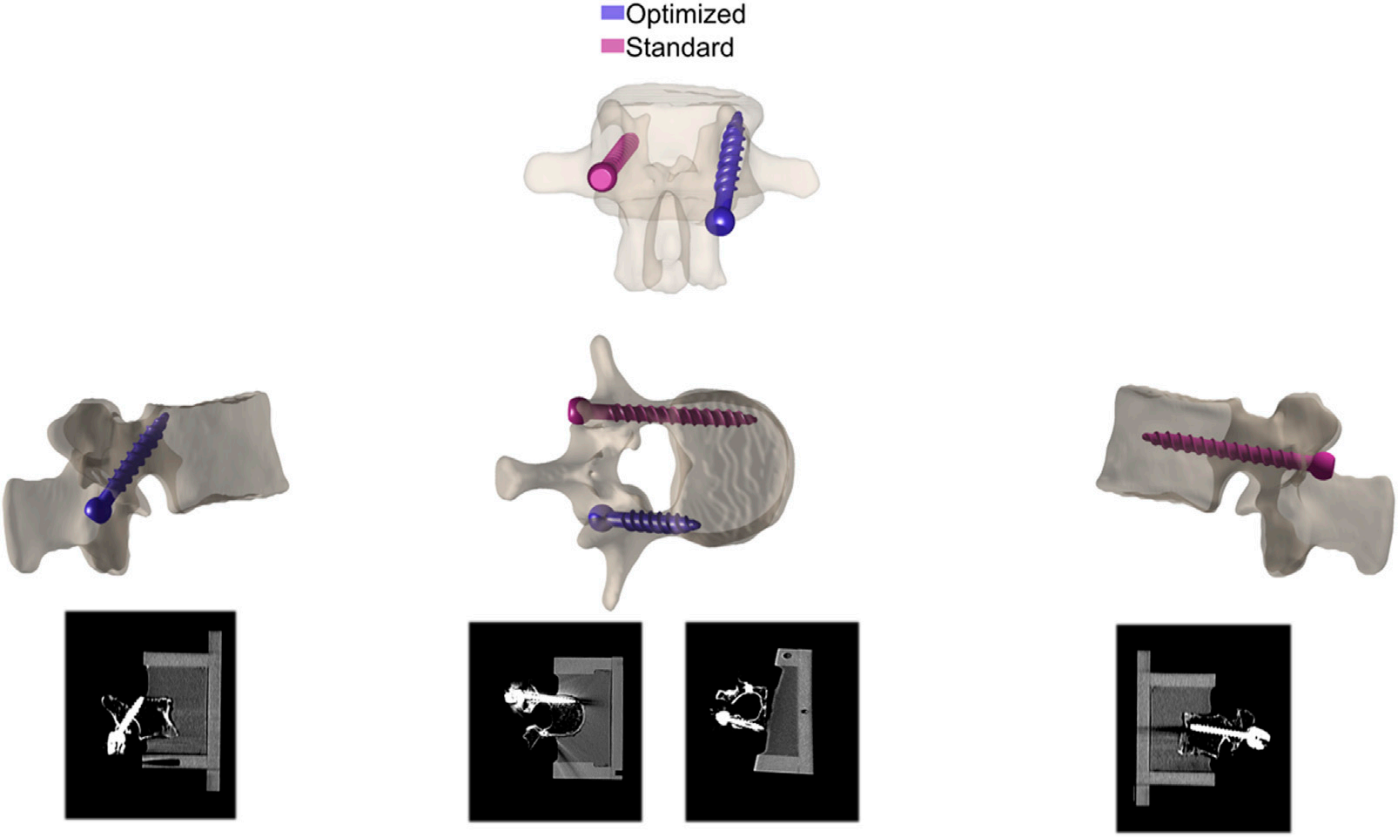
Screw position after insertion in traditional and optimized trajectories.

Computed tomography (CT) scans (SOMATOM Edge Plus, Siemens Healthcare GmbH, Erlangen, Germany) allowed assessment of vertebral bone integrity prior to screw insertion. Pedicle screws were inserted into both pedicles of each vertebral body with the help of a 3D-printed guide to ensure screw positioning according to planning. On one side, the pedicle screws were inserted with a traditional trajectory, i.e., parallel to the superior endplate and following the pedicle alignment (Zhang et al., 2006; Cann et al., 2015). On the other side, the inserted screws followed an optimized trajectory (Figure). In all cases, polyaxial pedicle screws (traditional trajectory: 1 × 5 × 40 mm, 2 × 5 × 45 mm, 4 × 6 × 45 mm, 6 × 6 × 50 mm, 5 × 6 × 55 mm, 1 × 6 × 65 mm, 1 × 7 × 40 mm, 3 × 7 × 45 mm, 2 × 7 × 55 mm; optimized trajectory: 1 × 5 × 30 mm, 2 × 5 × 35 mm, 1 × 5 × 40 mm, 1 × 5 × 45 mm, 1 × 5 × 50 mm, 1 × 6 × 25 mm, 5 × 6 × 30 mm, 2 × 7 × 35 mm, 4 × 7 × 40 mm, 3 × 7 × 45 mm, 2 × 7 × 55 mm, 2 × 7 × 60 mm) of the MUST pedicle screw system (Medacta International SA, Castel San Pietro, Switzerland) were used. The optimized screw was positioned within a spine and across all specimens in alternating pedicles (Figure 1).

Screw length and diameter were based on the size of the vertebral body and pedicle, respectively. After preparation, the anterior parts of the lumbar vertebral bodies were mounted in appropriate trays using Polymethylmethacrylate (PMMA; SCS-Beracryl D 28 powder and SCS-Veracryl D 28 liquid, Suter Kunststoffe AG, Fraubrunnen, Switzerland). The boxes were made of polyethylene terephthalate (PET) for CT transparency.

**FIGURE 2:**
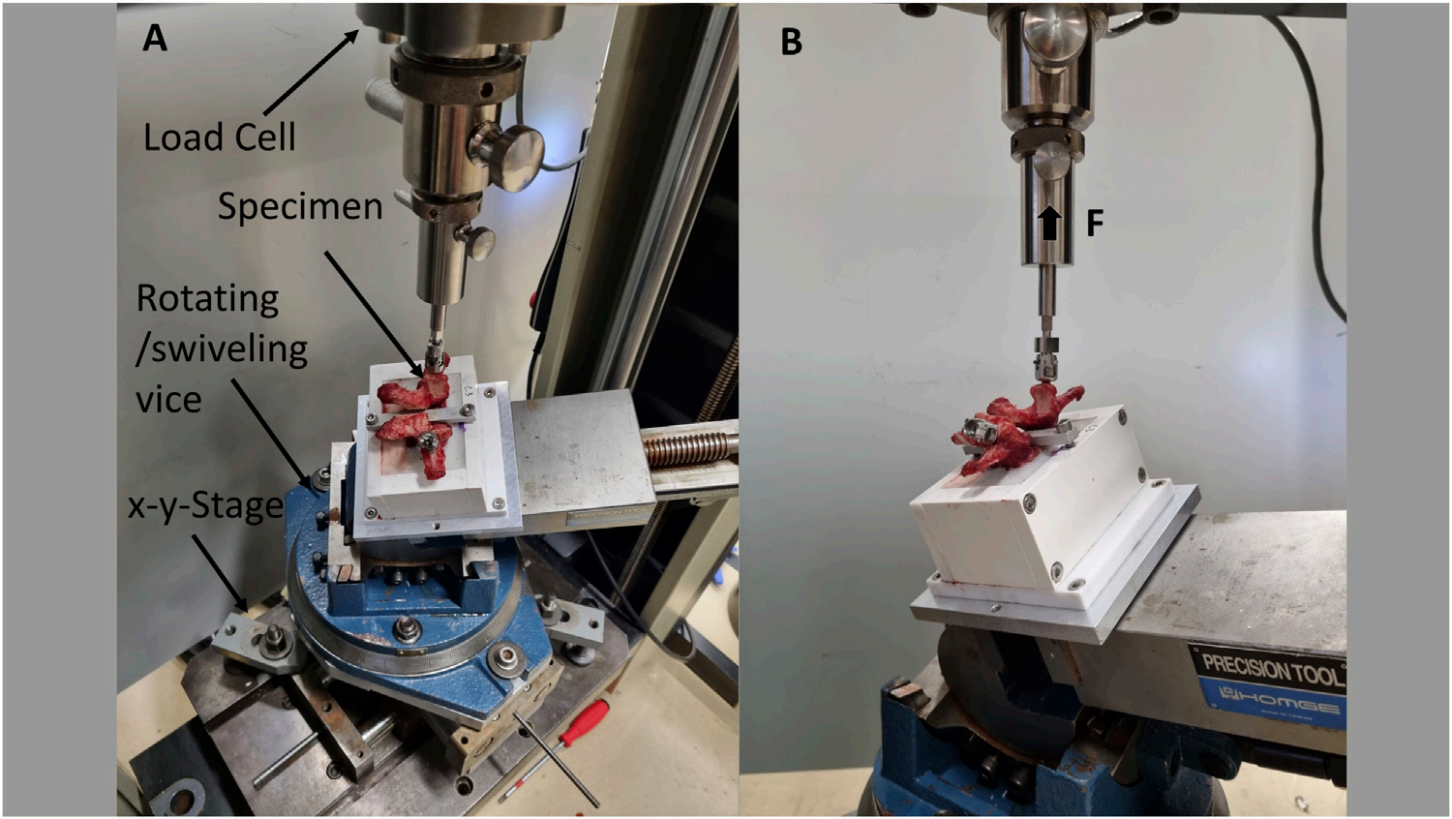
The setup for biomechanical testing: top view **(A)**, side view **(B)**: Two pedicle screws were inserted into the vertebral body and extracted using the uniaxial testing machine. The specimens were clamped in a universal 3-way tilting vice that was mounted on an X-Y stage that was free to move in the plane perpendicular to the direction of screw extraction. Angles were adjusted in all planes to achieve the calculated axial screw orientation.

The setup for testing specimens follows that of another study (Widmer et al., 2020). In a nutshell, to increase stability during testing, a metal plate was inserted through the vertebral foramina and attached to both sides of the boxes prior to testing. The specimens in the boxes were mounted in a universal 3-way tilting vice that was fixed on an X-Y table that was free to move in the plane perpendicular to the direction of screw extraction, and angles were adjusted in all planes to achieve the calculated axial screw alignment. A uniaxial tensile force was applied to the screw head using a universal testing machine (Zwick-Roell, Zwick GmbH, Ulm, Germany) (Figure 2). An Xforce load cell with a measurement accuracy of ± 0.5% over 100N, from the same supplier as the testing machine, was used. The screw extraction speed was set at 5 mm/min in accordance with the ASTM standard (Tolunay et al., 2015; Aycan et al., 2016; F543-17, A, 2017). A preload of 5N was applied to eliminate initial slack and improve alignment (Schmid et al., 2018).

### 2.3 Statistical analysis

The pull-out strength of each screw was defined as its resistance to axial loading and corresponded to the maximum of the experimentally recorded force-displacement curve. The strain energy was the area under the force-displacement curve from the beginning of the recording until the point at which pull-out strength was reached. Further, the estimated E-modulus within a screw resulted from its average value multiplied by the screw volume. Symmetry of anatomical and bone property aspects between the right and left sides of each vertebra was assumed. Therefore, the improvement in computed E-modulus and of the test results achieved through optimization resulted from the subtraction of the traditional value from the contralateral optimized value of the same vertebra. The diameter and length of the screws in the traditional trajectory group were compared to the screw sizes obtained with the optimization algorithm with a paired, non-parametric test (Wilcoxon signed-rank test). The significance level α was set to 0.05. Data were reported as median (25th percentile—75th percentile).

## 3 Results

### 3.1 Pull-out strength of optimized and traditional screw trajectory

**FIGURE 3:**
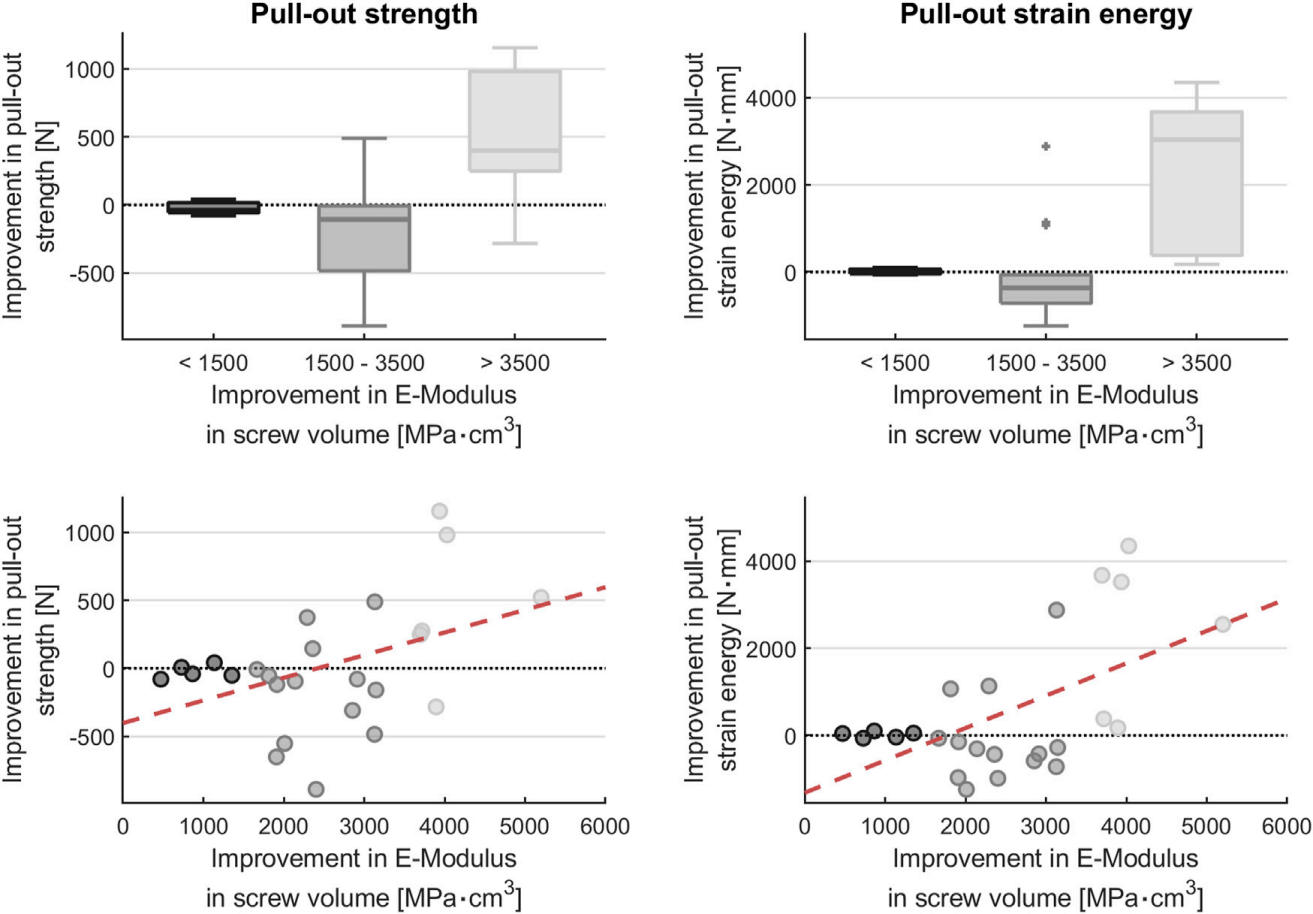
Plot of pull-out strength and strain energy as a function of improvement in average E-modulus times the screw volume: optimization increases the pull-out strength only when the optimizability is large (i.e., when the E-modulus difference is large). *Y*-axis: Pull-out strength improvement = difference between the pull-out strength of the optimized trajectory screws and the pull-out strength of the traditionally inserted screws in the contralateral pedicle. *X*-axis: Improvement in E-modulus in screw volume = difference in estimated bone E-modulus within the screw volume between optimized and contralateral traditionally inserted pedicle screws.

Figure 3 shows the pull-out strength and strain energy as a function of the improvement in E-modulus. The goal of the screw trajectory optimization algorithm was to insert the screws in areas of high bone E-modulus.

An improvement in pull-out strength and pull-out strain energy of the optimized screw trajectory compared to the traditional screw trajectory was only observed for values of average E-modulus times the screw volume greater than 3500  MPa cm^3^: the median pull-out strength improvement for this subset of screws was 399.0N (249.1–982.1N) and the median pull-out strain energy improvement was 3,034.3N mm (382.1–3,673.4N mm).

When the optimization resulted in values of 3500  MPa cm^3^ or less, optimization showed no benefit. For E-modulus values within the screw ranging between 1,500 and 3,500 MPa cm^3^, both median pull-out strength and median pull-out energy were lower than traditionally inserted screws, at −106.8N (−485.2 to −7.5N) and −363.7N mm (−715.4 to −63.4N mm), respectively. There was also no advantage for values below 1,500, with median pull-out strength and median pull-out energy of −39.9N (−58.3–16.5N) and 45.5N mm (−44.5–66.5N mm), respectively, lower than the traditionally inserted screws.

The median pull-out strength was 698.7N (361.2–1,526.2N) for pedicle screws with optimized trajectory. The median pull-out strength was 900.6N (415.9–1,352.4N) for traditionally inserted pedicle screws.

The median pull-out strain energy (N mm) was 1,244.8N mm (595.9–2962.5N mm) for pedicle screws with optimized trajectory. The median pull-out energy was 1076.5N mm (383.8–2512.4N mm) for traditionally inserted pedicle screws.

### 3.2 Screw characteristics of the optimized and traditionally inserted pedicle screws

**FIGURE 4:**
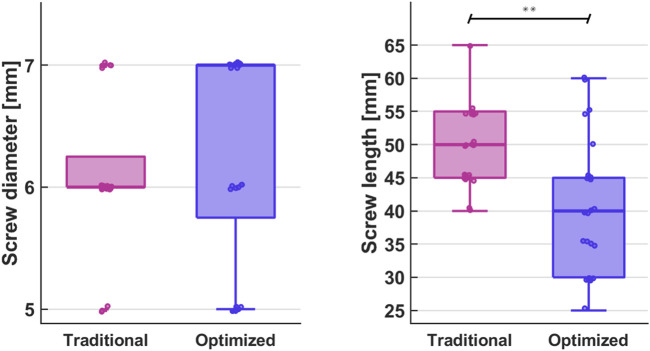
Comparison of screw diameter and length between traditionally inserted and optimized pedicle screws (**p < 0.001).

The median screw diameter of the optimized screws was 7 mm (range: 5.8–7 mm). The median screw diameter of the traditionally inserted screws was 6 mm (range: 6–6.3 mm) (Figure 4).

The median screw length of the optimized pedicle screws was 40 mm (range: 30–45 mm), which was significantly smaller than the median screw length of the traditionally inserted pedicle screws, which was 50 mm (45–55 mm), *p* < 0.001 (Figure 4).

## 4 Discussion

### 4.1 Summary and key outcomes

The main finding of the present study is that pedicle screw trajectory optimization is feasible. However, an increase in pull-out strength was only observed at a maximized difference in radiologically estimated E-modulus in screw volume (>3500  MPa cm^3^). When the difference between optimized and traditionally inserted pedicle screws was less than 3500 MPa cm^3^, the optimized trajectory showed no real advantage.

Fixation of lumbar motion segments with pedicle screws is the surgical gold standard for a variety of pathologies such as scoliosis, degenerative deformities, fractures, infections, and tumors (Wang et al., 2014; Tschugg et al., 2017; Chan et al., 2020; Perna et al., 2022). The stability of pedicle screws is known to depend on a variety of factors, including screw shape, diameter, length, thread shape, pitch width, difference between inner and outer cortex, bone mineral density, and screw trajectory (Phan et al., 2015; Delgado-Fernandez et al., 2017; Liu et al., 2020; Hsieh et al., 2021; Jarvers et al., 2021). In the search for an optimal solution, screw trajectories have been extensively studied clinically and biomechanically in recent years (Chang et al., 2021; Tai et al., 2022); comparing the most common methods such as the traditional trajectory, a modified trajectory directed caudally toward the antero-inferior margin of the vertebral body, and the cortical bone trajectory, which attempts to maximize contact between the screw thread and the cortical bone (Tai et al., 2022). In addition, numerous studies have been conducted using computer modeling to find ways to optimize pedicle screw positioning (Goerres et al., 2017; Knez et al., 2017; Mischler et al., 2020). Finding a trajectory that maximizes screw retention and minimizes the screw loosening risk and the associated pain and construct failure still does not exist. One reason might be that the optimizations in the latter studies follow trajectories that are closely aligned with standard clinical ones, suggesting that the incidence of screw loosening might be the same. To overcome this drawback, the trajectory optimization approach developed by Caprara et al. (Caprara et al., 2022) calculated and incorporated the E-modulus using Hounsfield Unit values from the input CT image, yielding promising results. Caprara et al. found that, compared to the standard clinical trajectory, the optimized screw instrumentation achieved better results in terms of pull-out strength and strain energy, with improvements of 26% and 75%, respectively. Interestingly, no differences in screw size were found between the two techniques, but the optimized screws were shorter on average than the surgeon’s choice. In the present study, the median pull-out strength and pull-out strain energy were comparable between the two methods. However, at differences in average E-modulus per screw volume of 3500  MPa cm^3^ and greater, considerably increased pull-out strength and pull-out strain energy were obtained for the optimized screws compared to the traditionally placed ones. Thus, the optimized trajectory appears to be superior to the standard trajectory when the optimization results in a considerable difference.

Looking at both screw size and screw length in this study, the traditionally inserted pedicle screws were larger and significantly longer than the screws that followed the optimized trajectory. The reason for this is that the optimization, which looked for the strongest bone in each case, sometimes resulted in unusual and therefore very short trajectories. This may be the reason why the results of the present study differ from those of the FE model used by Caprara et al. (Caprara et al., 2022), and it may also be the reason why the improvement in E-modulus needs to be maximized before the optimization becomes superior.

Therefore, it is worth considering whether the screw length should be given more weight in the algorithm or whether a minimum screw length should be included in the algorithm.

Overall, computational analysis methods have gained tremendous importance in the last decade as they facilitate and specify preoperative planning. Not only do they have the potential to improve the accuracy of surgical steps (e.g., pedicle screw insertion) (Farshad et al., 2017; Farshad et al., 2022), but they also consider and incorporate biomechanical aspects with the overall goal of improving clinical outcomes and minimizing long-term complications.

The results presented here should be considered as preliminary. It is intended that the algorithm will be further developed using screw length as an optimization criterion.

### 4.2 Limitations

In the present study, uniaxial pull-out tests were performed in order to draw conclusions about screw stability and thus the risk of screw loosening. However, these pull-out tests may not fully reflect the physiological loads to which the spine is subjected (Kueny et al., 2014), so the conclusions drawn from the results obtained must be evaluated with caution and may differ somewhat from reality. In particular, the contribution of both spinal musculature and gravity could not be assessed in this study. This is relevant for both in ambulatory loading settings and with regard to postoperative outcomes, as iatrogenic muscle damage during incisions prior to spinal instrumentation has been reported to have the potential to influence surgical outcomes (Tandon et al., 2018; Wang et al., 2023). Nevertheless, confirming the capacity of the proposed trajectories to increase screw retention and determining the characteristics of patients most likely to benefit is a step toward ensuring that a relevant clinical trial would be safe and effective.

In the present study, every effort was made to obtain the maximum number of lumbar specimens from a sample of cadavers with comparable age and sex characteristics. To minimize the impact of individual differences on the results of the studies, the design and placement of the screws were performed by the same team of researchers. It should also be noted that our results apply to the instrumentation material mentioned in the methods section, given that biomechanical properties such as fatigue resistance may differ between different instrumentation constructs (Massey et al., 2021).

A level-dependent analysis was not performed. We assumed geometric and mechanical symmetry in the vertebral bodies to compare the performance between standard and optimal screw positions within each vertebra. In general, we observed that the within-subject (i.e., between-level) differences in bone density were significantly less important than the between-subject (i.e., between-individual) differences.

Furthermore, the clinical applicability of the optimized trajectory needs to be further evaluated by experienced spine surgeons. Depending on the existing pathology and the required intervention, the optimized trajectory may prove inappropriate by compromising structures that should be protected. In addition, the algorithm does not explicitly take screw length into account. Incorporating this into the algorithm by weighing the length of the screw more than it is presently done may improve the pull-out strength. This could be investigated in future studies.

## 5 Conclusion

Optimizing pedicle screw trajectory is possible. However, the pull-out strength could only be improved with very high optimizations (E-modulus times screw volume >3,500 MPa cm^3^), because the optimization of the screw trajectory resulted in a reduction of the screw length. In the future, more emphasis should be placed on screw length.

## Data Availability

The datasets presented in this article are not readily available because Data can become available through a request and respective permission from the Spine Biomechanics Unit of the Balgrist University Hospital and the respective institutional ethical committee. Requests to access the datasets should be directed to JW, jonas.widmer@hest.ethz.ch.
