# Supplementary figures and images for: Bone density optimized pedicle screw insertion
Source: Front Bioeng Biotechnol. 2023 Oct 26;11:1270522. doi: 10.3389/fbioe.2023.1270522 (PMC10639121; doi:10.3389/fbioe.2023.1270522)

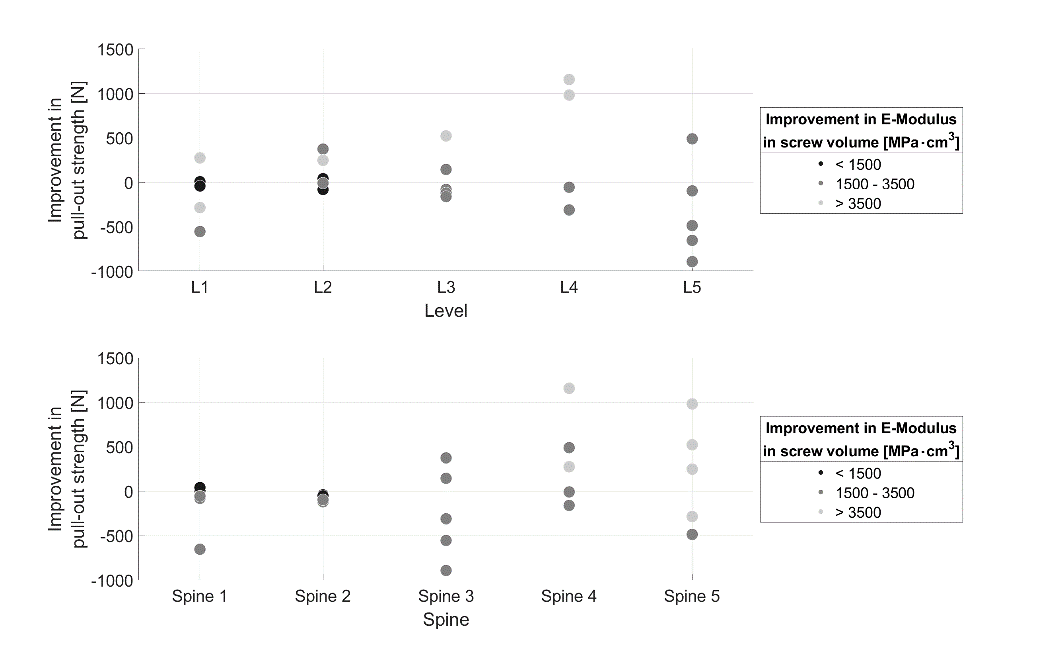

Supplement: Supplementary file 1 [file Image1.tiff]
